# Detection of perineural invasion in prostate needle biopsies with deep neural networks

**DOI:** 10.1007/s00428-022-03326-3

**Published:** 2022-04-21

**Authors:** Kimmo Kartasalo, Peter Ström, Pekka Ruusuvuori, Hemamali Samaratunga, Brett Delahunt, Toyonori Tsuzuki, Martin Eklund, Lars Egevad

**Affiliations:** 1 Department of Medical Epidemiology and Biostatistics, Karolinska Institutet, Stockholm, Sweden; 2 Institute of Biomedicine, University of Turku, Turku, Finland; 3 Faculty of Medicine and Health Technology, Tampere University, Tampere, Finland; 4 Aquesta Uropathology and University of Queensland, QLD, Brisbane, Australia; 5 Department of Pathology and Molecular Medicine, Wellington School of Medicine and Health Sciences, University of Otago, Wellington, New Zealand; 6 Department of Surgical Pathology, School of Medicine, Aichi Medical University, Nagoya, Japan; 7 Department of Oncology and Pathology, Karolinska Institutet, Radiumhemmet P1:02, Karolinska University Hospital, 171 76 Stockholm, Sweden

**Keywords:** Pathology, Artificial intelligence, Perineural invasion, Prostate cancer

## Abstract

The presence of perineural invasion (PNI) by carcinoma in prostate biopsies has been shown to be associated with poor prognosis. The assessment and quantification of PNI are, however, labor intensive. To aid pathologists in this task, we developed an artificial intelligence (AI) algorithm based on deep neural networks. We collected, digitized, and pixel-wise annotated the PNI findings in each of the approximately 80,000 biopsy cores from the 7406 men who underwent biopsy in a screening trial between 2012 and 2014. In total, 485 biopsy cores showed PNI. We also digitized more than 10% (*n* = 8318) of the PNI negative biopsy cores. Digitized biopsies from a random selection of 80% of the men were used to build the AI algorithm, while 20% were used to evaluate its performance. For detecting PNI in prostate biopsy cores, the AI had an estimated area under the receiver operating characteristics curve of 0.98 (95% CI 0.97–0.99) based on 106 PNI positive cores and 1652 PNI negative cores in the independent test set. For a pre-specified operating point, this translates to sensitivity of 0.87 and specificity of 0.97. The corresponding positive and negative predictive values were 0.67 and 0.99, respectively. The concordance of the AI with pathologists, measured by mean pairwise Cohen’s kappa (0.74), was comparable to inter-pathologist concordance (0.68 to 0.75). The proposed algorithm detects PNI in prostate biopsies with acceptable performance. This could aid pathologists by reducing the number of biopsies that need to be assessed for PNI and by highlighting regions of diagnostic interest.

**Supplementary Information:**

The online version contains supplementary material available at 10.1007/s00428-022-03326-3.

## Background

The identification of perineural invasion (PNI) by prostate carcinoma in prostate biopsies has been shown to be associated with poor outcomes [1, 2]. In the USA annually, approximately 1 million men undergo prostate biopsy and in various series PNI ranges from 7% to 33% of cases [1, 2]. The intuitive reason for the poorer prognosis of men with PNI is that the cancer has invaded the perineural space of at least one nerve, and it is via this route that the tumor is able to spread beyond the prostate. Despite increasing evidence of the prognostic significance of PNI, pathology reporting guidelines have, to date, not included PNI as a mandatory reporting element, although recently it has been included in prognostic guidelines for urologists [3, 4].

A possible reason for PNI not being included in prostate pathology reporting guidelines is that results from early studies, regarding the prognostic significance of PNI, were contradictory [5]. As a consequence, it would seem a reasonable conclusion that the reporting of PNI could not be justified [6]. More recent studies have indicated that the identification of PNI is clearly of prognostic significance, and there is a growing body of evidence to suggest that PNI should be routinely reported in prostate biopsies [5].

Workload issues are an on-going problem in pathology. Internationally, the number of pathologists in clinical practice is in decline, while the breadth and complexity of pathology reporting is increasing. This is especially so in the case of prostate biopsies, as the incidence of prostate cancer is increasing due to an increasing demand for informal/opportunistic screening for the disease in an aging population. The workload issue is further compounded by the increasing number of biopsy cores that are taken as part of random sampling of the prostate for cancer. Similarly, increasing numbers of cores are submitted from targeted biopsies that are designed to both diagnose and delineate the extent of malignancy. It is for this reason that recent initiatives have resulted in the development of artificial intelligence (AI) systems that have been designed to screen for cancer. The expectation here is that these will facilitate cancer diagnosis and assist pathologists in the routine screening of prostate cancer biopsies [7].

Recently, two studies, utilizing deep neural networks (DNN), have demonstrated AI systems to perform at an equivalent level to expert uro-pathologists in the grading prostate biopsies [8, 9]. These networks have been trained through the screening of thousands of sections of both benign and malignant biopsies, including a variety of cancer morphologies. A drawback that these current networks have is that they lack the capability to diagnose PNI. While PNI will be recognized as a malignant focus, its true nature is overlooked, which means that potentially useful prognostic information is lost. In this view, should AI play a future role in prostate cancer diagnosis, there is the need for systems to explicitly target relevant pathological features. Despite the obvious need to expand the role of AI systems in the diagnosis of prostate cancer, to the best of our knowledge, there are no studies that have systematically examined the performance of AI for the detection and localization of PNI and its reproducibility, when compared to expert pathologists.

This study is based upon a large series of prostate cancer biopsies accessioned prospectively as part of a trial to develop DNNs for the diagnosis of prostate cancer [10, 11]. Specifically, we have utilized a subset of cases to develop and validate the automatic detection of PNI in prostate biopsies, with the aim of demonstrating clinically useful diagnostic properties.

## Material and methods

### Study design and participants

This study is based upon biopsy cores from men who participated in the STHLM3 trial [10]. This was a prospective and population-based trial designed to evaluate a diagnostic model for prostate cancer. Patients in the trial were aged between 50 and 69 years, and cases were accrued between May 28, 2012 and December 30, 2014. Formal diagnosis of prostate cancer was by 10–12 core transrectal ultrasound–guided systematic biopsies.

### Slide preparation and digitization

**Fig. 1:**
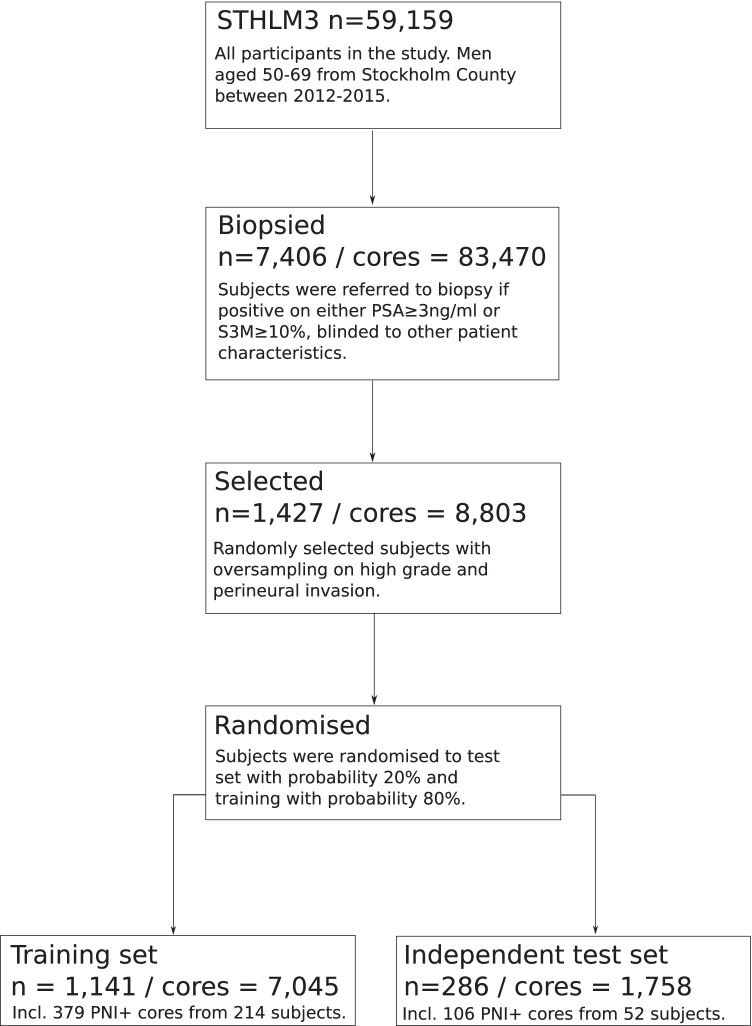
Patient flow diagram

The biopsy cores were formalin-fixed, stained with hematoxylin and eosin and mounted on glass slides. Histologic assessment was undertaken by the study pathologist (L. E.), and pathological features including cancer grade and PNI were entered into a database. We then randomly selected 1427 participants from which we retrieved 8803 biopsy cores. The selection was stratified by grade to include a larger sample of high-grade cancers and also included all cases containing PNI (Fig. 1; Supplementary Appendix 1). From these, we randomly assigned 20% of the subjects to a test set to evaluate PNI. The remaining 80% of subjects were used for developing and training the AI system.

### Slide annotations

Each slide containing PNI was re-assessed digitally by the study pathologist (L. E.), and all the regions of PNI were annotated pixel-wise using QuPath [12]. In total, there were 485 slides that contained at least one focus of cancer with associated PNI. Binary masks of the slides were generated, and they acted as pixel-wise ground truth labels for training and validation purposes (see Supplementary Appendix 1–2).

From the test set, all slides containing PNI (*n* = 106), according to the assessment of the study pathologist, as well as a random selection of non-PNI slides (*n* = 106), were re-assessed for the presence of PNI by three other experienced pathologists with a special interest in urological pathology (B. D., H. S., T. T.). The pathologists were blinded to the distribution of PNI in the biopsies and performed their assessments independently using Cytomine (Liège, Belgium) [13], as previously described [14].

### Deep neural networks

#### Patch extraction

To train the DNNs on PNI morphology, we extracted patches from each of the slides. Patch size was approximately 0.25 mm × 0.25 mm (see Supplementary Appendix 3), which was large enough to cover the size and shape of most of the nerves that showed infiltration by cancer. We evaluated different patch sizes within the training data with slightly lower performance (see Supplementary Appendix 4). To learn pixel-wise prediction we also extracted the corresponding region from the binary masks, which acted as labels.

#### Network architecture

Convolutional DNNs were used to classify patches (Xception) and to identify the regions in the biopsy where PNI was present (Unet) [15, 16].

For classification, we used soft voting (i.e., averaging probabilities) from an ensemble of 10 networks to generate final patch-wise probabilities. The highest probability for PNI among patches from a single core was used as a prediction score for classifying a core as PNI positive or negative at different operating points. Similarly, we used the highest predicted patch within a subject for subject-level classification.

For segmentation, i.e., pixel-wise prediction to identify the exact regions on each slide where the PNI was located, we first obtained pixel-wise predictions for each patch. We then re-mapped the predictions to the location from which each patch was extracted. We applied pixel-wise averaging across the slide for pixels with overlapping patches and over all networks in the ensemble to generate probabilities for each pixel to contain PNI. Finally, we used an a priori specified threshold to classify each pixel in the slide as PNI positive or negative (see Supplementary Appendix 3).

### Statistical analysis

The receiver operating characteristic (ROC) curve and the area under the ROC curve (AUC) were used to evaluate the performance of the algorithm. In addition, we analyzed four pre-specified operating points (the index test and three alternative positivity criteria) on which we have reported sensitivity, specificity, positive predictive value (PPV), negative predictive value (NPV), and accuracy. For evaluating pixel-wise segmentation, we used intersection over union (IoU). Specifically, we used all predicted positive and true positive pixels for each core to measure the core wise IoU, and then reported the average of these IoUs across all PNI positive cores. All analyses (except IoU) were undertaken both at individual core level and at subject level. Using a subset of the test set assessed by multiple pathologists, we evaluated the concordance between the AI and the pathologists in relation to inter-observer variability. Specifically, we calculated Cohen’s kappa for each pair of observers (including the AI), and then for each observer calculated the mean of the pairwise kappa values. Chi-^2^ test was used for comparisons of proportions.

All confidence intervals (CIs) were two-sided, with 95% confidence levels calculated from 1000 bootstrap samples. The DNNs and all analyses were implemented in Python (version 3.6.5) and TensorFlow (version 2.0.0) [17]. For the Unet implementation, we used the Python package segmentation_models with focal loss [18].

## Results

In this study of 1427 subjects, 266 (18.6%) were positive for PNI. The PNI positive men generally had higher serum prostate specific antigen (PSA) levels prior to biopsy, were more likely to have positive findings on digital rectal examination, and had cancers that were more often palpable and of higher grade (Table 1). From these subjects 8803 slides were examined, of which 485 (5.5%) were positive for PNI.

**Table 1:** Top: Patient characteristics. There were 11 patients (8 of 266 PNI positive men) on whom we could not retrieve clinical information. Bottom: Slide characteristics. There was no missing information. † The values in parentheses are the Gleason scores

	PNI+ men (*n* = 266)	PNI− men (*n* = 1161)	*p*-value
	No. (%)	No. (%)	(chi^2^ test)
Age			0.28
< 49 yr	2 (0.8)	4 (0.3)	
50–54 yr	13 (5.0)	89 (7.7)	
55–59 yr	35 (13.6)	170 (14.7)	
60–64 yr	83 (32.2)	303 (26.2)	
65–69 yr	119 (46.1)	564 (48.7)	
≥ 70 yr	6 (2.3)	28 (2.4)	
PSA			< 0.001
< 3 ng/mL	46 (17.8)	271 (23.4)	
3–5 ng/mL	78 (30.2)	531 (45.9)	
5–10 ng/mL	71 (27.5)	261 (22.5)	
≥ 10 ng/mL	63 (24.4)	95 (8.2	
Digital rectal examination			< 0.001
Abnormal	109 (42.2)	119 (10.3)	
Normal	149 (57.8)	1039 (89.7)	
Prostate volume			0.0025
< 3 5 mL	138 (53.5)	487 (42.1)	
35–50 mL	74 (28.7)	382 (33.0)	
≥ 50 mL	46 (17.8)	289 (25.0)	
Cancer length			< 0.001
No cancer	0 (0.0)	176 (15.2)	
0–1 mm	4 (1.6)	169 (14.6)	
1–5 mm	13 (5.0)	335 (28.9)	
5–10 mm	37 (14.3)	170 (14.7)	
> 10 mm	204 (79.1)	308 (26.6)	
ISUP grade†			< 0.001
Benign	0 (0.0)	176 (15.2)	
ISUP 1 (3 + 3)	40 (15.5)	522 (45.1)	
ISUP 2 (3 + 4)	96 (37.2)	248 (21.4)	
ISUP 3 (4 + 3)	58 (22.5)	89 (7.7)	
ISUP 4 (4 + 4, 3 + 5, 5 + 3)	25 (9.7)	68 (5.9)	
ISUP 5 (4 + 5, 5 + 4, 5 + 5)	39 (15.1)	55 (4.7)	
	PNI+ slides (*n* = 485)	PNI−slides (*n* = 8318)	*p*-value
	No. (%)	**No. (%)**	(chi^2^ test)
Cancer length			< 0.001
No cancer	0 (0.0)	4712 (56.6)	
0–1 mm	69 (14.2)	1121 (13.5)	
1–5 mm	134 (27.6)	1547 (18.6)	
5–10 mm	170 (35.1)	698 (8.4)	
> 10 mm	112 (23.1)	240 (2.9)	
ISUP grade†			< 0.001
Benign	0 (0.0)	4712 (56.6)	
ISUP 1 (3 + 3)	97 (20.0)	1892 (22.7)	
ISUP 2 (3 + 4)	130 (26.8)	680 (8.2)	
ISUP 3 (4 + 3)	81 (16.7)	321 (3.9)	
ISUP 4 (4 + 4, 3 + 5, 5 + 3)	85 (17.5)	469 (5.6)	
ISUP 5 (4 + 5, 5 + 4, 5 + 5)	92 (19.0)	244 (2.9)	

**Fig. 2:**
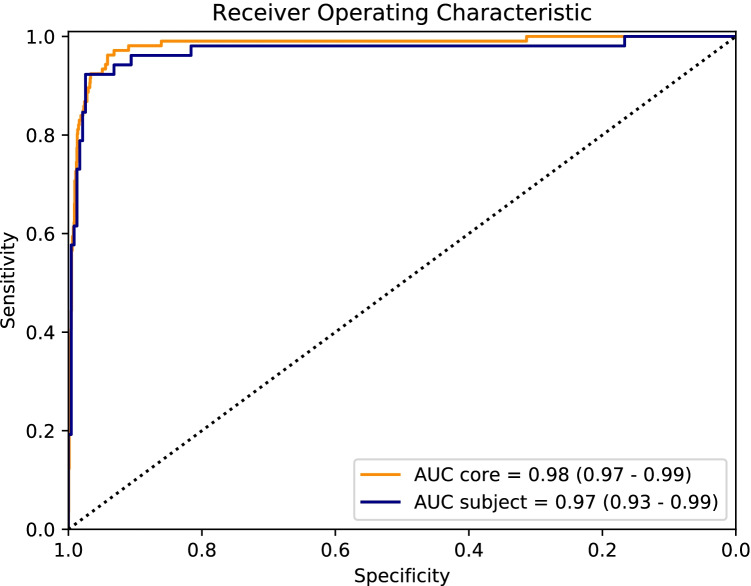
Performance of the network to discriminate between PNI and non-PNI in individual cores (orange) and in subjects (blue). The curves are based on *n* = 1758 (*n* = 106 positive) cores and *n* = 286 (*n* = 52 positive) subjects. The values in parentheses are confidence intervals

The AUC for discriminating between PNI positive and negative was 0.98 (CI: 0.97–0.99) for individual slides (PNI positive = 106, PNI negative = 1652), and 0.97 (CI: 0.93–0.99) for subjects (PNI positive = 52, PNI negative = 234) (Fig. 2). Sensitivity and specificity, positive and negative predictive values, and accuracy at the index test’s operating point and three alternative operating points are shown in Table 2, both at the level of individual cores and at a subject level.

**Table 2:** Diagnostic metrics for the network. The operating points are alternative thresholds for positivity. The point marked (index test) is the value on which the algorithm is intended to be used, and the other three show the diagnostic properties of the model if a different sensitivity to specificity relationship is preferred

	Operating point	Sensitivity	Specificity	PPV	NPV	Accuracy
Cores	0.99	0.82	0.98	0.78	0.99	0.97
	0.95 (index test)	0.87	0.97	0.67	0.99	0.97
	0.90	0.92	0.96	0.60	0.99	0.96
	0.85	0.92	0.95	0.54	0.99	0.95
Subjects	0.99	0.92	0.96	0.84	0.98	0.95
	0.95 (index test)	0.94	0.91	0.69	0.99	0.91
	0.90	0.96	0.85	0.60	0.99	0.87
	0.85	0.96	0.84	0.57	0.99	0.86

*PPV* positive predictive value, *NPV* negative predictive value

**Fig. 3:**
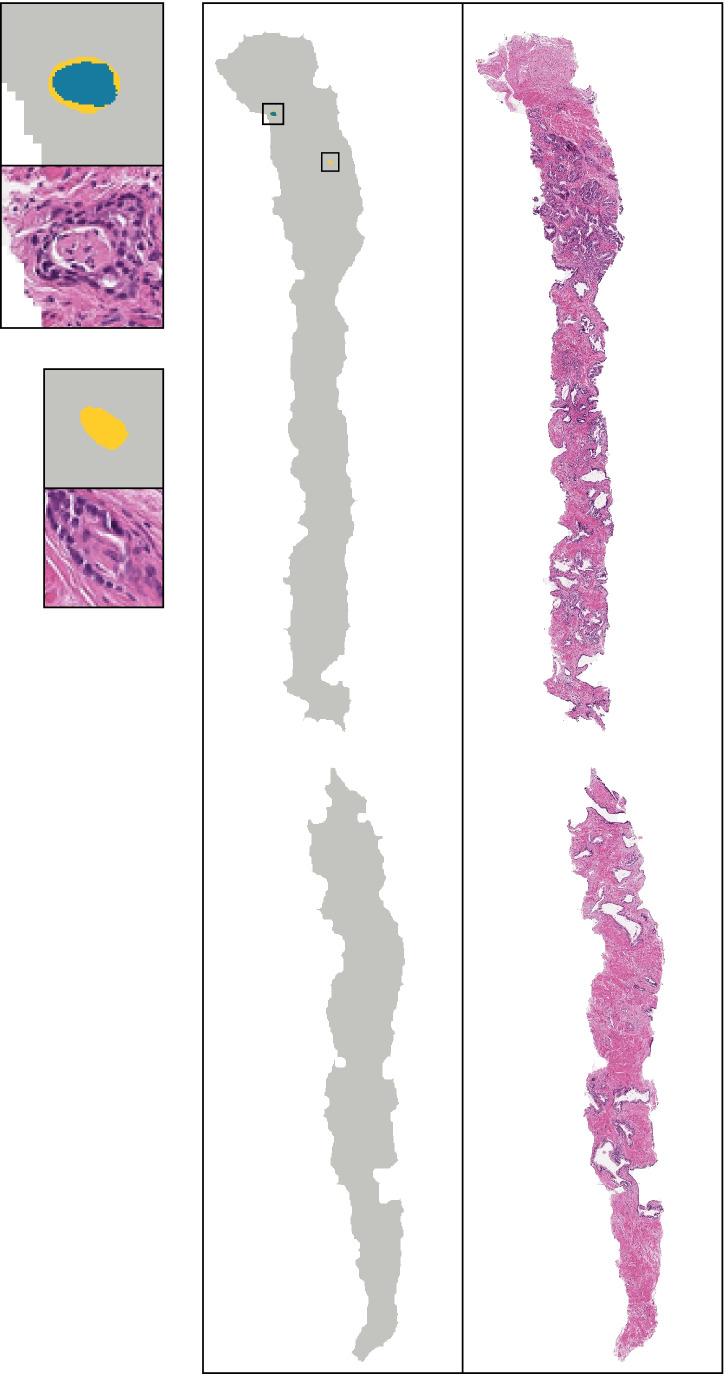
Illustration of PNI segmentation on the biopsy core with IoU (0.51) closest to the overall mean IoU (0.50) reported. The H&E stained biopsy **(**right) and the corresponding predicted pixel-wise classification and ground truth (middle), and two highlighted regions (left) are shown. The regions both annotated by the pathologist and classified as positive by the network (i.e., the intersection) are colored blue, and the regions not annotated but still classified positive by the network are yellow. In this example, there were no regions annotated but not classified as positive. All pixels positive by either the pathologist or the network form the union (i.e., the denominator in the IoU)

The estimated mean IoU across slides was 0.50 (CI: 0.46–0.55), which measures pixel-wise agreement between the study pathologist’s annotation of PNI and the pixels classified as positive by the algorithm. For reference, Fig. 3 shows the individual PNI positive slide in the test set with IoU closest to the mean IoU.

**Fig. 4:**
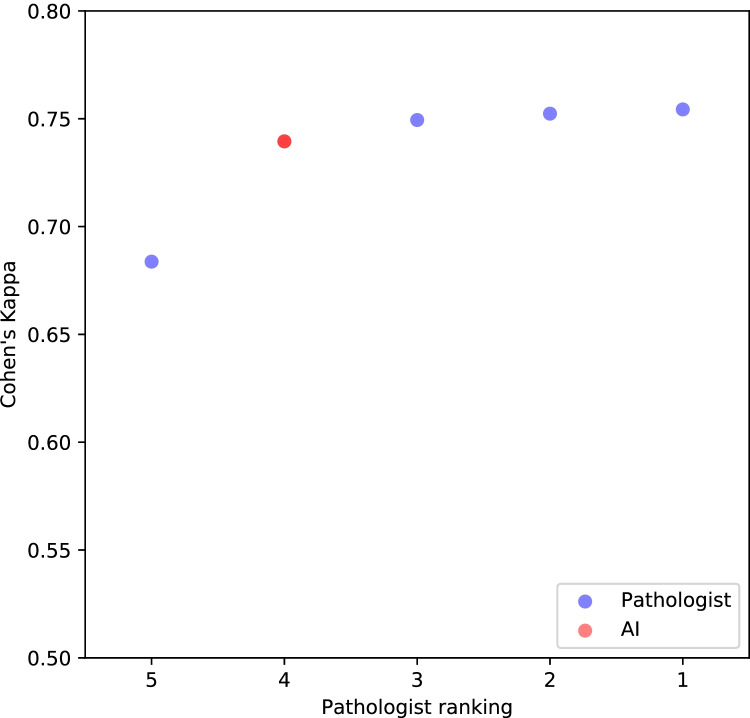
Cohen’s kappa for pathologists (blue) and the AI (red) evaluated on the test set (*n* = 212). The data points represent the mean pairwise kappa for each of the observers, including the AI, compared with the others. The observers are ranked according to the kappa value.

On the subset of the test set assessed by multiple pathologists (PNI positive = 106, PNI negative = 106, according to initial assessment), comparable performance was observed irrespective of which pathologist provided the reference standard (AUC of 0.97, 0.95, 0.94, and 0.93; see Supplementary Figure S4). When evaluated for concordance in terms of mean pairwise Cohen’s kappa (Fig. 4), the AI (0.740) was within the range of the pathologists (0.684 to 0.754).

**Fig. 5:**
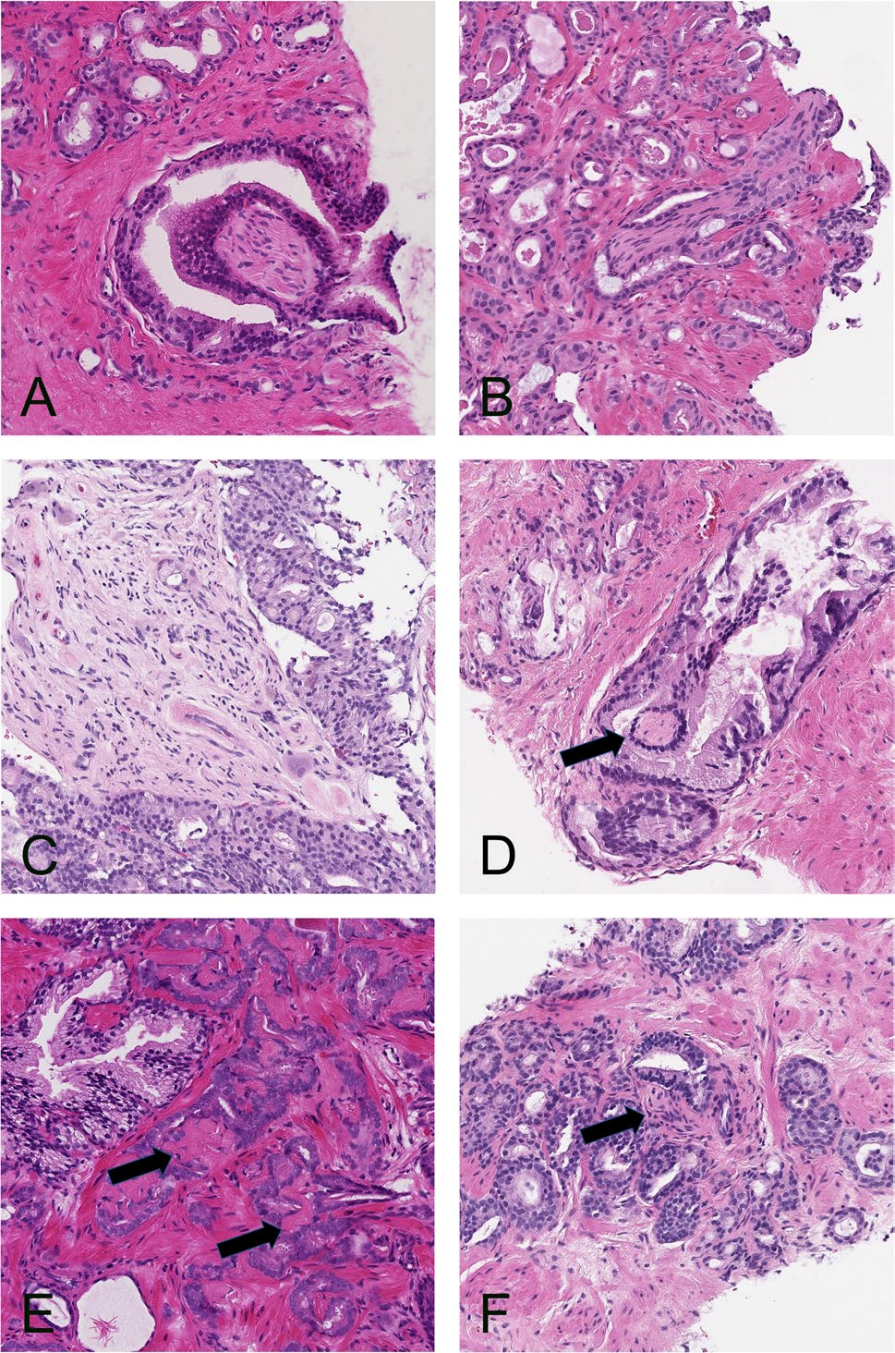
**A** and **B**. PNI that was correctly identified by AI. **C** and **D**. PNI that was reported false negative by AI. In **C** there is invasion of a ganglion, and in **D** there is a minimal entrapped nerve that resembles stroma (arrow). **E and F**. Structures that were reported false positive for PNI by AI. In **E**, there is mucinous fibroplasia resembling nerves (arrows), and in **F**, there is reactive stroma which mimics a nerve (arrow). All microphotographs show hematoxylin and eosin stains at 20× lens magnification

Cases where AI made a false positive or false negative diagnosis of PNI were reviewed. Reasons for a false positive diagnosis included mucinous fibroplasia, reactive stroma, and bundles of smooth muscle (Fig. 5). The reasons for a false negative diagnosis included small nerve bundles in a reactive stroma, small entrapped nerves resembling stroma, and invasion of ganglia (Fig. 5).

## Discussion

Even though the propensity of prostate cancer to invade perineural spaces is well known, it is only comparatively recently that it has been shown to be independently associated with poor outcome. The presence of PNI in needle biopsies appears to predict outcome after radical prostatectomy [2, 19, 20], and in accordance with this, urology practice guidelines support the reporting of PNI [4]. The assessment of needle biopsies for the presence of PNI is tedious and hampered by inter-observer variability [14], which itself may have contributed to previous confusion relating to the prognostic significance of the parameter. Currently, pathology reporting guidelines, issued by both international bodies and jurisdictional pathology authorities, do not include PNI as a required element [21–23]. In view of the increasing evidence of the utility of PNI detection as a prognostic parameter, this is likely to change [5, 22]. In addition, there is evidence that quantitative measures of PNI may have prognostic relevance, such as the diameter of PNI foci in radical prostatectomy specimens [24] and the extent of PNI in preoperative core biopsies [25]. If implemented in routine reporting, such time-consuming quantitative assessment would be facilitated by the assistance of AI. In some cases, identification of PNI may be problematic, with foci of infiltration of the perineural space being difficult to distinguish from stromal bundles or collagenous micronodules situated adjacent to malignant glands. In this context, DNNs may assist in the detection of PNI, as they appear to reach a more consistent level of performance when compared to the subjective observations of diagnostic pathologists. Since DNNs are consistent in their decisions and are easily distributed, they also have potential value as a teaching tool.

In this study, we have demonstrated a novel deep learning system which we have shown to detect PNI in prostate cancer biopsies with high AUC. Use of the system may also assist pathologists by suggesting regions of interest for the detection of PNI in a biopsy slide. The main strength of this study is that we have identified, digitized, and annotated all foci of PNI reported in more than 80,000 cores from all men who underwent biopsy as part of the STHLM3 trial. Since this trial was based upon a randomized population-based selection of men, there is a strong probability that the tumors sampled have displayed a broad spectrum of morphologies. This in turn suggests that the results may be generalized, and the diagnostic algorithms developed in this study are applicable to other populations. Further strengths of the study are that our AI system was validated by a large independent test set and that the pathology of all the cases in the series was evaluated and reported by a single specialist prostate pathologist.

The main limitation of the study is that, due to the high cost of digitizing the samples, we could not include biopsies from all the subjects that participated in the STHLM3 trial in this PNI study. We felt that this was untenable due to the relatively low value that would have been added to the study by including all the numerous cores with no tumor and those with only low-grade cancers. Another limitation is that the review of the cases was done by a single observer. External review of PNI negative cases would have been at least as important as the review of PNI positive cases, but it would not have been a realistic study design to request a multiobserver review of more than 8000 slides. We have previously shown that the kappa statistics for interobserver reproducibility of PNI assessment were as high as 0.73 (including the current study pathologist) without previous training [14]. Furthermore, PNI was not verified by immunohistochemistry as a previous study demonstrated this to be of limited value [14].

The selection of cases for study was random, but cases were stratified by grade to include sufficient high-grade cancers in the series. This was considered necessary as high-grade prostate cancers are relatively infrequently encountered in screening trials. This makes it difficult to interpret the predictive values, which depend on the prevalence of positive and negative cases. For example, if we had not over-sampled positive cases, the already high NPV would likely be even higher and the somewhat low PPV would likely have been even lower. Even if the PPV appears low — as approximately half of the predicted positive slides do not contain PNI — it should be noted that in the series, PNI was not present in most cases. Despite this, the assessment of only those slides which were predicted as positive would result in a substantial reduction in work for the reporting pathologist. Importantly, the AUCs (sensitivity and specificity) were not confounded by artificial oversampling of positive cases.

Another limitation in the study was a difficulty in the interpretation of IoU. In this study, we chose to define intersection and union as all the pixels of PNI in a slide as a single object. This did not consider relative sizes of PNI within a specific slide. For example, given a slide containing a large and a small PNI focus, one would obtain a higher IoU by fully detecting the large focus and fully failing to identify the small focus, rather than by partially detecting both foci. We would argue that the latter would still be more desirable when using the system as a diagnostic aid, but this is not captured by the IoU metric. Finally, we have not tested the algorithm on external data. We know from other studies involving whole slide images that one can expect some loss in performance on an external test set. This loss of performance is most likely due to differences in laboratory staining protocols, subjectivity in the assessment of pathology, and potentially the use of different types of scanners to digitize the biopsy cores. To overcome these limitations, it is preferable to include a large sample in training the networks, specimens from different laboratories, a variety of types of image scanners, and a wide range of prostate tumors showing differing morphologies.

Deep neural networks have shown excellent results in the automation of the grading of cancers in prostate biopsies. An important limitation, however, is an inherent lack of flexibility. DNNs only perform the task that they are trained on and do not directly reveal information relating to other findings. As we move towards a fully automated assessment of biopsies, we will need to develop systems that can address the interpretation of features that are additional to diagnosis and grading. All potential tasks do not need to be incorporated in a single DNN but can be implemented through several separate models, with each performing a single task. The evolving digital revolution of pathology will undoubtedly give rise to abundant data that can be utilized for training such specific models.

## Conclusion

This study has demonstrated that deep neural networks can screen appropriately for perineural invasion by cancer in prostate biopsies. This has the potential to reduce the workload for pathologists. Application of such systems will also allow for automatic interpretation of large datasets, which can be utilized to increase knowledge relating to the relationship between perineural invasion by prostate cancer and poor patient outcome.

## Supplementary information

Below is the link to the electronic supplementary material.

Supplementary file1 (PDF 943 kb)

## Declarations

The study was approved by the Regional Ethic Review Board, Stockholm (2012/572-31/1, 2018/845-32).
