# Supplementary material for: Detection of perineural invasion in prostate needle biopsies with deep neural networks
Source: Virchows Arch. 2022 Apr 21;481(1):73–82. doi: 10.1007/s00428-022-03326-3 (PMC9226086; doi:10.1007/s00428-022-03326-3)
Supplement: Supplementary file 1 — Supplementary file1 (PDF 943 kb) [file 428_2022_3326_MOESM1_ESM.pdf]

Supplementary Appendix:

Detection of Perineural Invasion in Prostate Needle Biopsies with Deep Neural Networks

Kimmo Kartasalo, Ph.D.[1], Peter Ström, Ph.D.[1], Pekka Ruusuvuori, Ph.D.[2,3],
Hemamali Samaratunga, FRCPA[4], Brett Delahunt, M.D.[5],
Toyonori Tsuzuki, M.D.[6], Martin Eklund, Ph.D.[1], Lars Egevad, M.D.[7]

1. Department of Medical Epidemiology and Biostatistics, Karolinska Institutet, Stockholm, Sweden.
2. Institute of Biomedicine, University of Turku, Turku, Finland.
3. Faculty of Medicine and Health Technology, Tampere University, Tampere, Finland.
4. Aquesta Uropathology and University of Queensland, Brisbane, QLD, Australia.
5. Department of Pathology and Molecular Medicine, Wellington School of Medicine and Health Sciences, University of Otago, Wellington, New Zealand.
6. Department of Surgical Pathology, School of Medicine, Aichi Medical University, Nagoya, Japan.
7. Department of Oncology and Pathology, Karolinska Institutet, Stockholm, Sweden.

Appendix 1: Data acquisition 1
Appendix 2: Pre-processing 2
Appendix 3: Neural Networks 3
    Patch extraction 3
    Classification network 3
    Segmentation network 3
    Hardware and software 4
Appendix 4: Complementary results 4
References 7

Appendix 1: Data acquisition

We used the STHLM3 register to select subjects whose biopsies we digitized. The first 500 men who were diagnosed in the STHLM3 trial were selected. This contributed the majority of slides, n=5,662. Since it was a screening-by-invitation trial, there were relatively few high-grade cases in this selection. To increase the coverage of the more rare high-grade cases and their associated morphologies, we included all subjects (their positive cores and a randomly selected negative core) with at least one core graded as Gleason Score (GS) 4+4 or 5+5, and a random selection of 497 subjects with at least one core graded 3+3. To increase the sample diversity of benign prostate tissue, we included 139 randomly selected cancer-free subjects from which we included one randomly selected core each. At this stage we had selected 1,289

participants from which we retrieved 8,571 biopsy cores. Finally, we included all cores in the STHLM3 register which had PNI reported which concluded a total of 8,803 slides from 1,427 subjects. In some of these, the study pathologist did not manage to reproduce the PNI finding. This could be due to difficult to judge cases or the difference of using microscopy (the original assessment) and digital pathology (the annotation of PNI for this study). These slides were included as negative cases.

The study pathologist (L.E.) subsequently highlighted each lesion of PNI pixel-wise in the digitized cores that were reported to contain PNI. This was done in the open source pathology software QuPath.[1] From these annotations we created binary masks, i.e. images indicating the presence or absence of PNI for each pixel of the associated whole slide images. The binary masks acted as pixel-wise ground truth labels when training and evaluating the networks.

There were two types of scanners used for digitizing the slides: Hamamatsu C9600-12 scanner running NDP.scan software v. 2.5.86 (Hamamatsu Photonics, Hamamatsu, Japan) and Aperio ScanScope AT2 scanner running Aperio Image Library v. 12.0.15 software (Leica Biosystems, Wetzlar, Germany). At full-resolution (20X) the pixel sizes were 0.45202 µm (Hamamatsu) and 0.5032 µm (Aperio). The RGB images were stored at 8-bits per channel in NDPI (Hamamatsu) and SVS (Aperio) format.

Appendix 2: Pre-processing

The scanned images typically contain two consecutive sections from a single biopsy core. The annotation of PNI was done on one of these two sections arbitrarily chosen by the study pathologist. To only include the annotated section in the training, we semi-automatically removed the unannotated one. In the cases where the annotated section was located in its entirety within one half of the image, and the unannotated section within the other half, we retained the image half containing the annotated section. Otherwise, removal of the unannotated section was performed manually.

Images were downsampled by a factor of 16 and converted from RGB to grayscale in accordance with the NTSC standard by calculating the weighted sum 0.2989 x R + 0.5870 x G + 0.1140 x B for each pixel. The tissue was segmented using Laplacian filtering, followed by thresholding the absolute magnitude of the resulting response using Otsu's method.[2] This resulted in binary masks indicating the tissue regions. For further details see Supplementary of Ström et al.[3]

To create label masks, that is, masks with values 0 for background, 1 for non-PNI tissue, and 2 for PNI, we exported binary masks from QuPath for each unique region annotated by the pathologist. We also stored the pixel coordinates from which they were extracted. Finally, we removed the complementary section in the mask as described above.

Appendix 3: Neural Networks

Patch extraction

For classification, we cropped patches with dimensions of 598 x 598 pixels at the highest resolution (20X) of the whole slide images, corresponding to roughly 300 µm x 300 µm. The patches were then labeled as PNI positive if they contained at least one pixel of PNI based on the binary masks.

For segmentation, we only used the slides positive for PNI and cropped patches of size 512 x 512 pixels at 20X from them, corresponding to 250 µm x 250 µm. The reason for using a different patch size than for classification was to match the different network architectures' default input sizes by factors of 2. In addition to cropping the tissue, we also cropped the corresponding region from the binary mask. These patches of the masks acted as the target variable when training the model for pixel-wise segmentation of PNI.

Since most parts of the image are background, we only considered patches with at least half of the pixels containing tissue according to the tissue mask. Patches were systematically cropped from all parts of the whole slide images containing tissue, with 50% overlap between adjacent patches. Finally, the patches were stored on disk as .jpg using JPEG compression with 80% quality.

Classification network

The deep neural network (DNN) used for classification of PNI on the patch level was Xception.[4] Ten models were trained in an identical way (except for randomization in initiating the model weights and the sampling and sample order of patches), and an average of these models' predictions was used as the final patch level prediction. For slide-level prediction, the maximum over the predictions of all patches from a slide was taken to represent the entire slide. Similarly, slide-level predictions were aggregated into subject-level predictions by taking the maximum over all slides from a given subject.

For training the DNNs we used a batch size of 8, randomly initiated weights, partial class balancing via oversampling of the rare positive class (2 PNI-negative patches sampled per one PNI-positive patch) to counter the extreme class imbalance of the data, binary crossentropy loss, Adam as optimizer with learning rate 0.001 and with other parameters set to default values, and trained for 100 epochs.[5] We augmented the data with random horizontal flips and random rotations of 90, 180, and 270 degrees.

Segmentation network

For segmentation we applied Unet on the patches.[6] Similarly to the classification models, we used an ensemble of 10 DNNs. The prediction patches (i.e. pixel-wise predictions for PNI for an image patch) were mapped to their original positions in the image to produce a prediction image corresponding to each whole slide image. A threshold was used to generate a binary version of the prediction image.

For training the DNNs we used a batch size of 8, randomly initiated weights, partial class balancing via oversampling of the rare positive class (4 PNI-negative patches sampled per

one PNI-positive patch) to counter the extreme class imbalance of the data, Adam as optimizer with a learning rate of 0.0001 and other parameters set to default values, focal binary loss and trained for 20 epochs.[7] We augmented the data with random horizontal flips and random rotations of 90, 180, and 270 degrees. Pixel-wise probabilities were averaged across the models in the ensemble and scaled from a floating-point range of [0, 1] to an unsigned 8-bit integer range of [0, 255]) to store the information as heatmap images. Finally, a pre-specified threshold of 75 was used to classify each pixel as positive. The reason that this was lower than a corresponding probability of 0.5 was to err on the side of sensitivity rather than specificity, since we argue that it is better to highlight additional potential foci rather than too few in the case of pixel-wise visualization.

Hardware and software

Computations were performed on a graphics processing unit (GPU) cluster (Tampere Center for Scientific Computing, Tampere, Finland) equipped with 28 x Tesla V100 and 32 x Tesla P100 GPUs (Nvidia, Santa Clara, CA, USA) distributed on 15 nodes. The GPUs were running Nvidia driver 440.64, CUDA 10.0.130 and cuDNN 7.6.0. The nodes were equipped with either a 20-core Xeon E5-2640 v4 or a 24-core Xeon Silver 4116 CPU (Intel, Santa Clara, CA, USA) and 254 GB, 385 GB or 785 GB of RAM. Each node was equipped with a local SSD disk. We used 32-bit floating point precision for GPU computation.

We used OpenSlide (v. 3.4.1) via the Python interface (v. 1.1.1) to access the images.[8] MATLAB R2017b (The MathWorks, Natick, MA, USA) and python were used to create the necessary masks for pixel-wise labeling of tissue and PNI. DNNs were implemented in Python 3.6.4 and TensorFlow (v 2.0.0).[9]

Appendix 4: Complementary results

We evaluated a number of design choices on training data. The evaluation was performed on a fixed validation split of 20% of the training data, using the remaining data for training. The split was performed on subject level.

For classification we compared several DNN architectures: Inception V3, Xception, ResNet, InceptionResNetV2, NASNet, EfficentNet (B7).[10–15] Of these, Inception V3 and Xception generally had the best performance across various hyperparameter settings and appeared most reliable in terms of few severe drops in performance (data not shown). Xception exhibited slightly better performance than Inception V3. A comparison between the two architectures is shown in Figure S1.

Moreover, we evaluated several patch sizes and resolution combinations (see three well performing combinations in Figure S1). In addition, we evaluated different strides for extracting patches and observed that a stride of 299 pixels (that is, 50% overlap between patches of 598 x 598 pixels), was superior to a stride of 150 pixels (data not shown).

For segmentation, we compared several backbones for Unet: VGG16, ResNet, ResNeXt, Inceptionv3, and EfficientNet (B7).[16] Inception V3 was chosen based on consistent high

performance on various sampling strategies and learning rates (data not shown). We also compared several loss functions (see Figure S2 and S3).

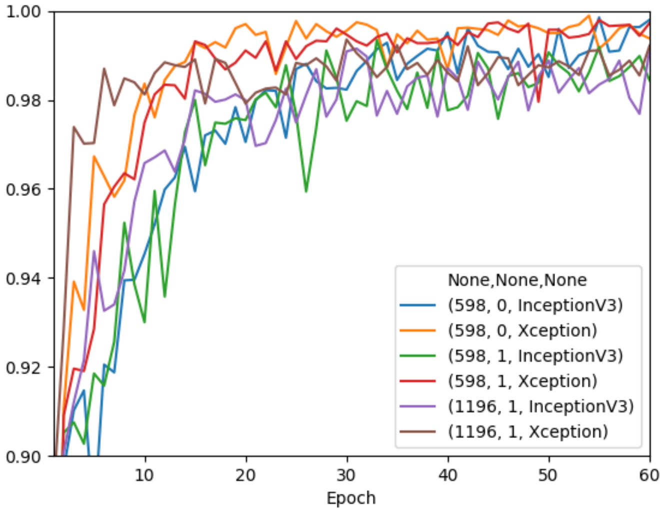

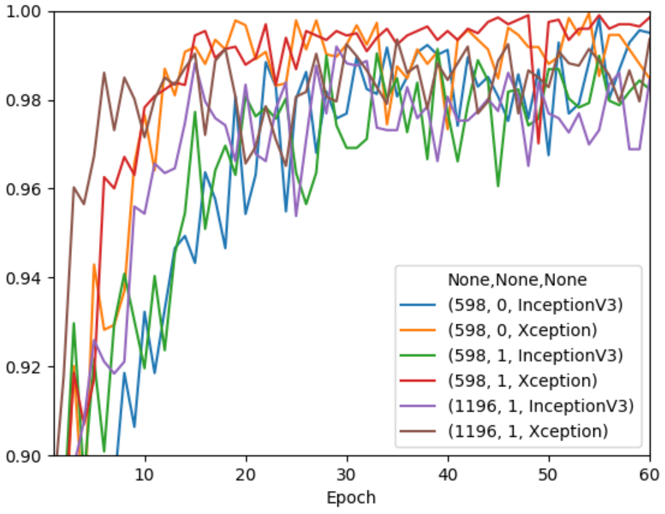

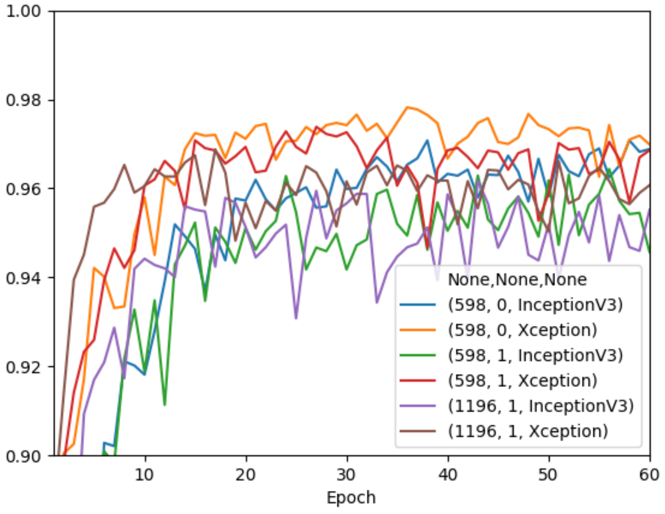

Figure S1: AUC for PNI classification using different patch sizes (598 and 1196 pixels), resolution (20X and 10X), and architecture (Inception V3 and Xception). From top: slide level, subject level, and patch level.

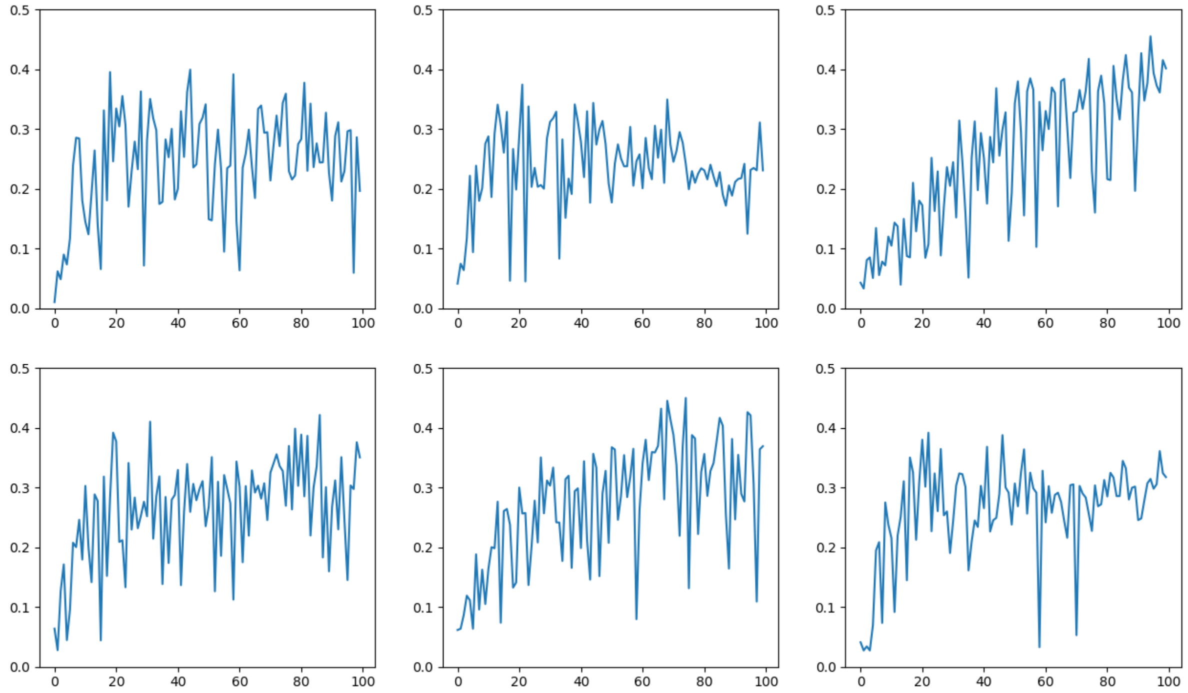

Figure S2: Intersection over union on patch level for various loss functions**. (First row; from left)** binary focal Jaccard loss, binary crossentropy, and Jaccard loss. **(Second row; from left)** binary focal dice loss, dice loss, and binary focal loss. The x-axis is number of epochs.

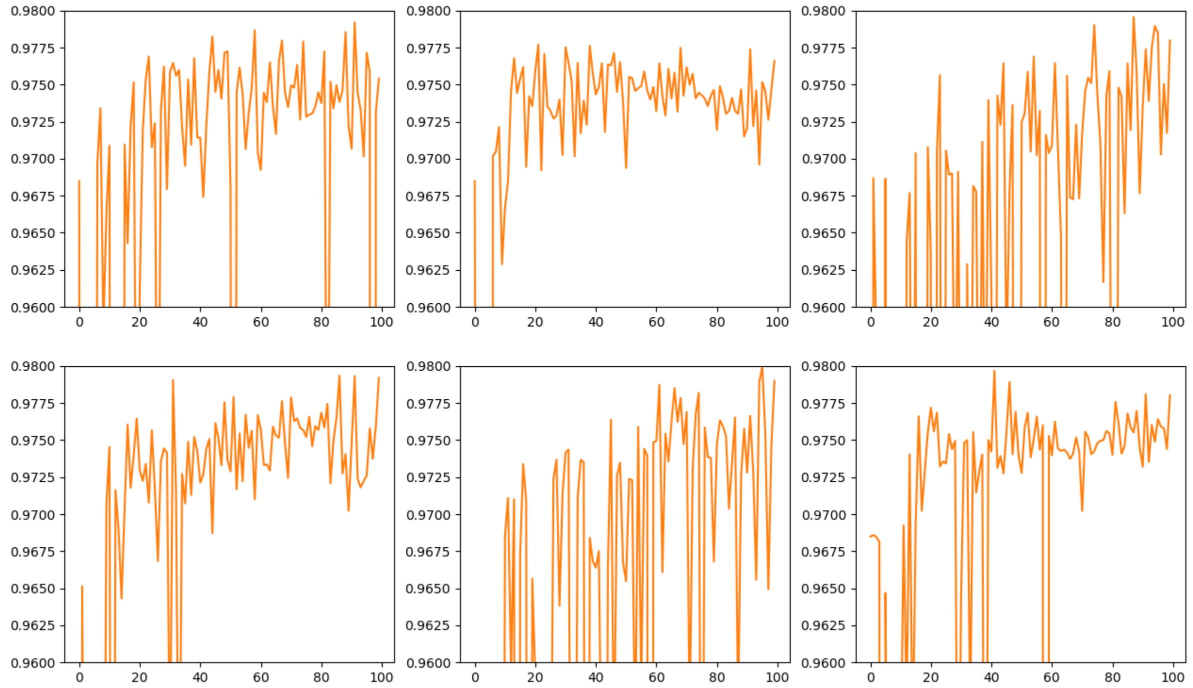

Figure S3: Accuracy on patch level for various loss functions**. (First row; from left)** binary focal Jaccard loss, binary crossentropy, and Jaccard loss. **(Second row; from left)** binary focal dice loss, dice loss, and binary focal loss. The x-axis is number of epochs.

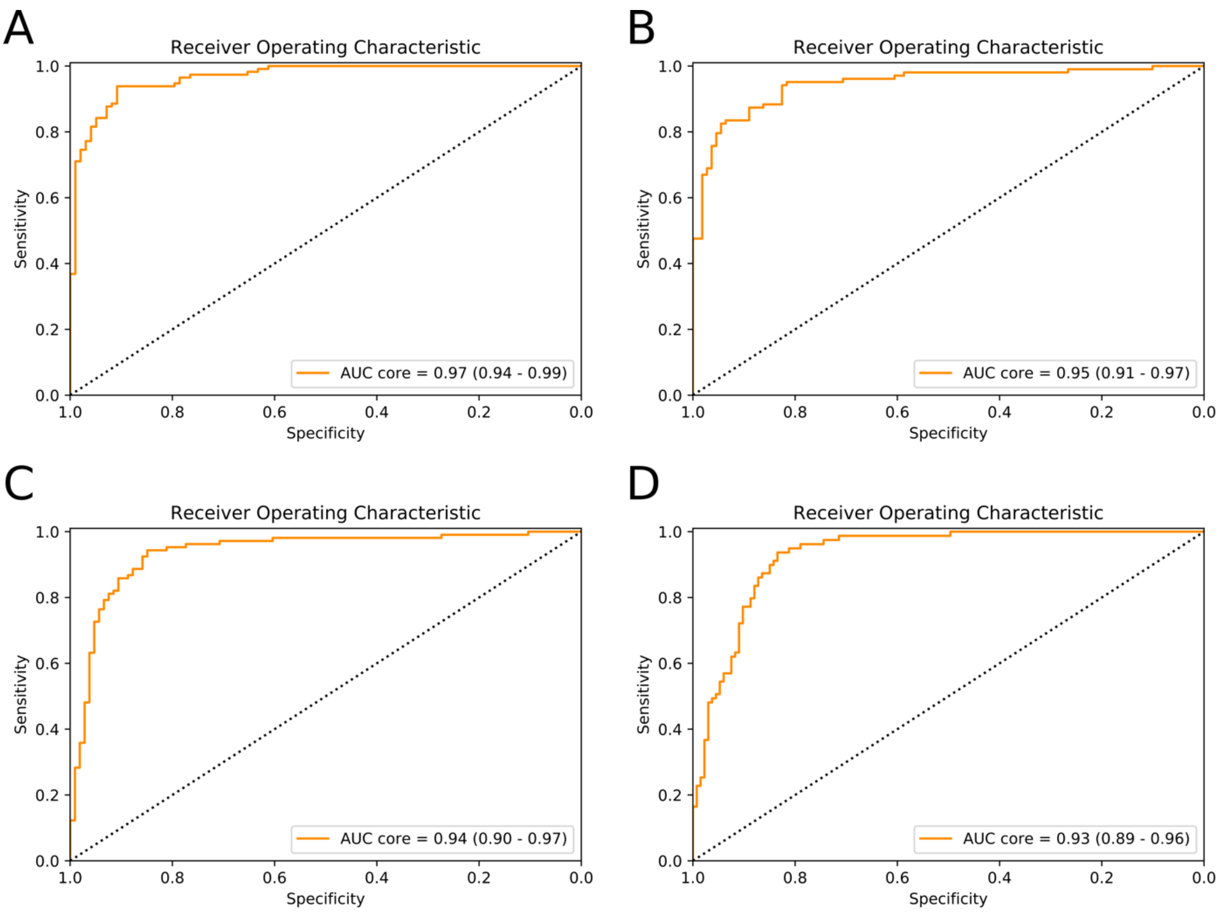

Figure S4: Receiver operating characteristics analysis of the AI model's performance in discriminating between PNI and non-PNI cores, evaluated using four reference standards, each set by a different pathologist **(A-D)**. The analysis was performed on a subset of the test set (N=212, 106 positive and 106 negative for PNI according to original pathology reports) independently assessed by the four pathologists. The area under the curve (AUC) is shown for each reference standard, with 95% confidence intervals in parentheses. The reference standard used in **(A)** was set by the same pathologist (L.E.) whose diagnoses were used for AI training.

References

1. Bankhead, P. *et al.* QuPath: Open source software for digital pathology image analysis. *Sci. Rep.* **7**, 16878 (2017).
2. Otsu, N. A Threshold Selection Method from Gray-Level Histograms. *IEEE Trans. Syst. Man. Cybern.* **9**, 62–66 (1979).
3. Strom, P. *et al.* Artificial intelligence for diagnosis and grading of prostate cancer in biopsies: a population-based, diagnostic study. *Lancet. Oncol.* (2020). doi:10.1016/S1470-2045(19)30738-7
4. Chollet, F. Xception: Deep Learning with Depthwise Separable Convolutions. *2017 IEEE Conference on Computer Vision and Pattern Recognition (CVPR)* (2017).

doi:10.1109/cvpr.2017.195
5. Kingma, D. P. & Ba, J. Adam: a method for stochastic optimization. CoRR abs/1412.6980 (2014). (2014).
6. Ronneberger, O., Fischer, P. & Brox, T. U-Net: Convolutional Networks for Biomedical Image Segmentation. *Lecture Notes in Computer Science* 234–241 (2015). doi:10.1007/978-3-319-24574-4_28
7. Lin, T.-Y., Goyal, P., Girshick, R., He, K. & Dollar, P. Focal Loss for Dense Object Detection. *IEEE Trans. Pattern Anal. Mach. Intell.* **42**, 318–327 (2020).
8. Goode, A., Gilbert, B., Harkes, J., Jukic, D. & others. OpenSlide: A vendor-neutral software foundation for digital pathology. *J. Pathol.* (2013).
9. Martín Abadi *et al.* TensorFlow: Large-Scale Machine Learning on Heterogeneous Systems. (2015).
10. Szegedy, C., Vanhoucke, V., Ioffe, S., Shlens, J. & Wojna, Z. Rethinking the Inception Architecture for Computer Vision. *Proceedings of the IEEE Computer Society Conference on Computer Vision and Pattern Recognition* **2016-Decem**, 2818–2826 (2016).
11. Chollet, F. Xception: Deep Learning with Depthwise Separable Convolutions. *2017 IEEE Conference on Computer Vision and Pattern Recognition (CVPR)* (2017). doi:10.1109/cvpr.2017.195
12. He, K., Zhang, X., Ren, S. & Sun, J. *Deep Residual Learning for Image Recognition*. (2016).
13. Szegedy, C., Ioffe, S., Vanhoucke, V. & Alemi, A. A. *Inception-v4, Inception-ResNet and the Impact of Residual Connections on Learning*. *Thirty-First AAAI Conference on Artificial Intelligence* (2017).
14. Zoph, B., Brain, G., Vasudevan, V., Shlens, J. & Le Google Brain, Q. V. *Learning Transferable Architectures for Scalable Image Recognition*. *openaccess.thecvf.com*
15. Tan, M. & Le, Q. V. EfficientNet: Rethinking model scaling for convolutional neural networks. in *36th International Conference on Machine Learning, ICML 2019* **2019-June**, 10691–10700 (International Machine Learning Society (IMLS), 2019).
16. Simonyan, K. & Zisserman, A. Very deep convolutional networks for large-scale image recognition. in *3rd International Conference on Learning Representations, ICLR 2015 - Conference Track Proceedings* (International Conference on Learning Representations, ICLR, 2015).
